# Changes in Brain Function and Structure After Self-Administered Home Photobiomodulation Treatment in a Concussion Case

**DOI:** 10.3389/fneur.2020.00952

**Published:** 2020-09-08

**Authors:** Linda L. Chao, Cody Barlow, Mahta Karimpoor, Lew Lim

**Affiliations:** ^1^Departments of Radiology & Biomedical Imaging and Psychiatry & Behavioral Sciences, University of California, San Francisco, San Francisco, CA, United States; ^2^VA Advanced Imaging Research Center, San Francisco VA Health Care System, San Francisco, CA, United States; ^3^Vielight Inc., Toronto, ON, Canada

**Keywords:** traumatic brain injury (TBI), photobiomodulation, home treatment, cognition, neuroimaging

## Abstract

Traumatic brain injury (TBI) is a common neurological disorder among athletes. Although there are no widely accepted treatments for TBI, new investigational approaches, such as photobiomodulation (PBM), are being tested. PBM is a light therapy that uses red to near-infrared (NIR) light to stimulate, heal, and protect tissue that has been injured or is at risk of dying. Benefits following transcranial PBM treatments in animal models of acute TBI and a small number of chronic TBI patients have been reported. However, the human PBM TBI studies published to date have been based on behavioral assessments. This report describes changes in behavioral and neuroimaging measures after 8 weeks of PBM treatments. The subject was a 23-year professional hockey player with a history of concussions, presumed to have caused his symptoms of headaches, mild anxiety, and difficulty concentrating. He treated himself at home with commercially available, low-risk PBM devices that used light-emitting diodes (LEDs) to emit 810-nm light pulsing at 10 or 40 Hz delivered by an intranasal and four transcranial modules that targeted nodes of the default mode network (DMN) with a maximum power density of 100 mW/cm^2^. After 8 weeks of PBM treatments, increased brain volumes, improved functional connectivity, and increased cerebral perfusion and improvements on neuropsychological test scores were observed. Although this is a single, sport-related case with a history of concussions, these positive findings encourage replication studies that could provide further validation for this non-invasive, non-pharmacological modality as a viable treatment option for TBI.

## Introduction

Traumatic brain injury (TBI) is a common and devastating health problem: An estimated 1.6–3.8 million concussions occur annually, and up to 10% of athletes suffer a concussion in any given sports season (1). There is presently no widely accepted treatment for TBI, although investigational approaches are being tested (2). Photobiomodulation (PBM) is a form of light-based therapy that exposes neural tissue to a low fluence of light (from <1 to >20 J/cm^2^), most commonly in the red to near-infrared (NIR) wavelengths (3). Preclinical studies suggest that transcranial PBM has beneficial effects in animal models of acute TBI (4–9). Cadaver studies show that transcranial applications of NIR light can penetrate to a depth of about 40 mm in the brain (10). Thus, PBM can be applied to humans non-invasively. While a few reports have described transcranial PBM treatments in chronic TBI patients (11–14), these studies were uncontrolled, the measures have been based on behavioral assessments, and the PBM treatments were administered in the laboratory or hospital. Although wider acceptance of PBM as a treatment modality for TBI is pending larger controlled studies, objective neuroimaging measures can provide data to help validate this modality as a viable treatment option for TBI. This report is the first study to use neuroimaging to investigate brain changes after 8 weeks of transcranial and intranasal PBM treatment in a subject with a history of concussion.

## Materials and Methods

**Figure 1:**
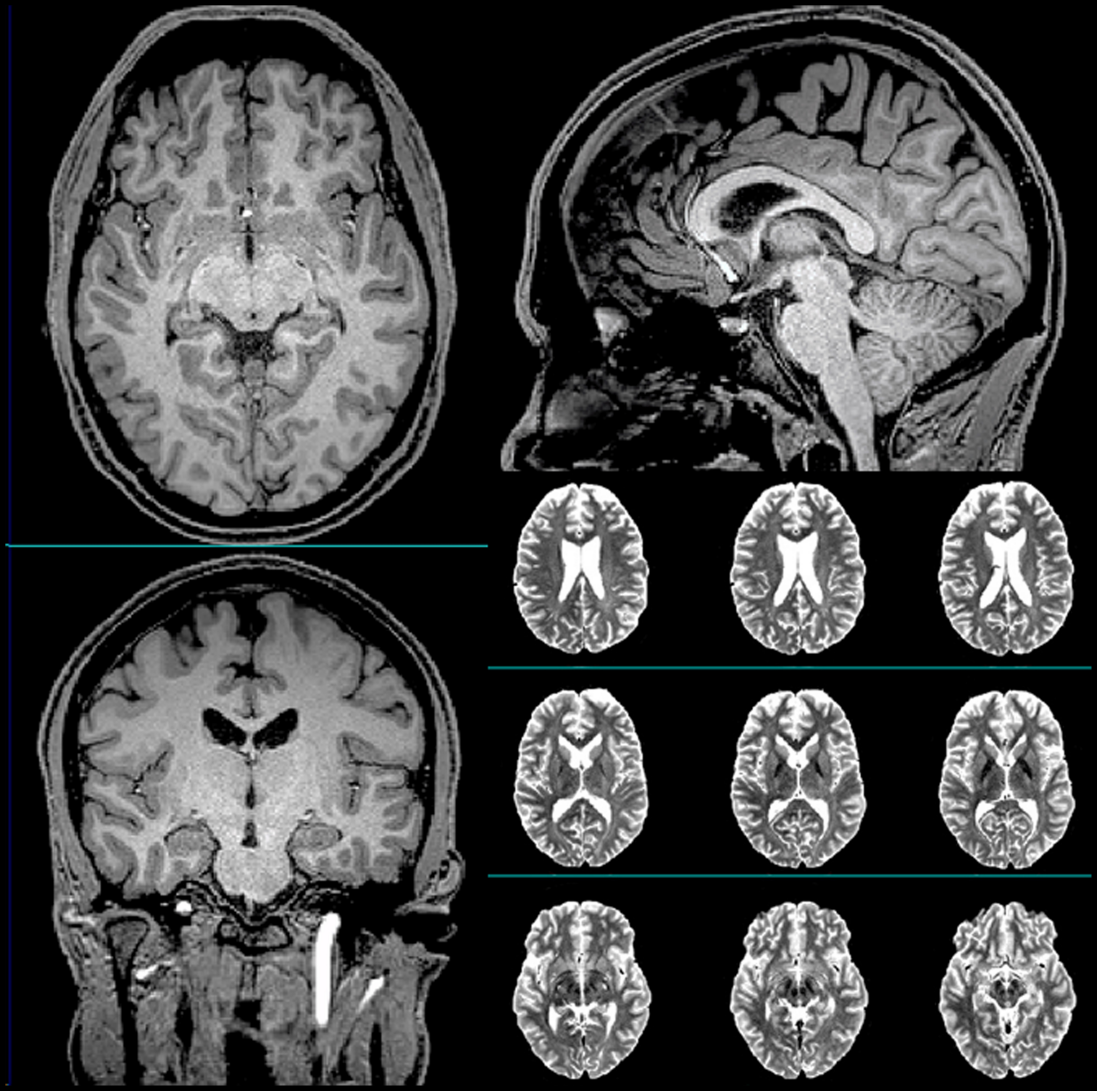
Representative T1-weighted (axial, sagittal, and coronal) and T2-weighted (axial) anatomical scans of the subject acquired at baseline.

The subject was a 23-year-old White non-Latino male with no family history of neuropsychiatric disorders and a favorable psychosocial background. His medical history included seasonal allergies, anisocoria, a condition where the pupils of the eyes differ in size, a fractured vertebra, a broken wrist, and six “documented” concussions. The subject was a professional hockey player, and all his injuries were hockey-related. Table 1 summarizes the timeline of the subject's history of concussion. The subject had never been diagnosed with mild TBI (mTBI). His clinical head computed tomography (CT) and magnetic resonance imaging (MRI) exams had been negative (see Figure 1 for representative baseline MRIs). The subject had experienced concussion-related memory gaps and feeling dazed; however, he had never lost consciousness. Thus, he was classified as a “possible TBI” by the Ohio State University TBI screen (15).

**Table 1:** Timeline of the subject's concussions, PBM treatments, and testing.

**Date**	**Event**
September, 2013	1st documented concussion, sustained during a hockey game
April, 2015	2nd documented concussion, sustained during a hockey game
May, 2017	3rd documented concussion, sustained during a hockey game
November, 2017	4th documented concussion, sustained during a hockey game
May, 2018	5th documented concussion, sustained during a hockey game
February, 2019	6th documented concussion, sustained during a hockey game
March 4, 2019	Baseline MRI, HIT-6, and neuropsychological testing
March 4, 2019	Commenced transcranial/intranasal PBM treatments with Vielight Neuro Gamma
March 10, 2019	Subject advised to administer transcranial/intranasal PBM treatments with Vielight Neuro Alpha device after developing mild headaches
March 20, 2019	Subject advised to alternate using Vielight Neuro Alpha and Gamma devices after headaches resolved
April 20, 2019	Subject advised to continue transcranial/intranasal PBM treatments with just the Vielight Neuro Alpha device
May 1, 2019	Post-treatment MRI, HIT-6, and neuropsychological testing
July 1, 2020	Follow-up HIT-6 assessment

The subject approached Vielight, Inc., in Toronto, Canada about their PBM devices after his last concussion left him with a desire to “improve his mental sharpness.” Prior to starting treatment, the subject's symptoms included headaches [six-item Headache Impact Test (HIT-6) (16) score = 76], mild anxiety, difficulty concentrating, and an inability to maintain attention. The subject reported previously trying acupuncture, nutritional supplements, and hyperbaric oxygen treatments for his condition. This report was undertaken as an exercise to observe potential improvements in the subject's cognitive and brain function after PBM treatments.

Vielight provided the subject with two non-thermal, non-laser light-emitting diode (LED) devices as wellness devices for his mental acuity. Vielight requested the subject to undergo neuroimaging and cognitive assessment by the first author so that observations could be made on whether objective neuroimaging data can support changes in cognition. The subject consented to this. The PBM devices used are considered non-regulated under “General Wellness: Policy for Low Risk” published by the Food and Drug Administration in September 2019.

The Vielight Neuro Alpha delivers 810-nm light pulsing at 10 Hz, 50% duty cycle; the Vielight Neuro Gamma delivers 810-nm light pulsing at 40 Hz, 50% duty cycle. Both devices have four transcranial and one intranasal LED modules designed to target nodes of the default mode network (DMN) (17), a group of strongly interconnected brain regions (18) that include the medial prefrontal/anterior cingulate cortex (targeted by the anterior transcranial LED), posterior cingulate cortex/precuneus (targeted by the central posterior transcranial LED), lateral parietal cortex (targeted by two lateral posterior transcranial LEDs), ventromedial prefrontal and entorhinal cortex, and hippocampus (targeted by the intranasal LED).

The posterior transcranial LEDs have a power of 100 milliwatts (mW) each; the anterior transcranial LED, 75 mW; and the intranasal LED, 25 mW. Each posterior transcranial LED has a power density of 100 mW/cm^2^; the anterior transcranial LED, 75 mW/cm^2^; and the intranasal LED, 25 mW/cm^2^. The beam spot size of each LED is about 1 cm^2^. The energy delivered by posterior transcranial LEDs is 60 joules (J); anterior transcranial LED, 45 J; and intranasal LED, 15 J. The energy density of the posterior transcranial LEDs is 60 J/cm^2^; anterior transcranial LED, 45 J/cm^2^; and intranasal LED, 15 J/cm^2^. Both the PBM devices, programmed to shut off automatically after 20 min, deliver 240 J per 20-min treatment session.

Table 1 summarizes the timeline of the subject's assessments and PBM treatments. After baseline assessments, the subject began home PBM treatments every other day with the Vielight Neuro Gamma device (pulsing 810 nm at 40 Hz). This recommendation was based on a report of cognitive improvements after participants had used the Vielight Neuro Gamma device (19). However, the subject developed mild headaches after 1 week of using the Neuro Gamma device. Consequently, Vielight advised him to switch to the Neuro Alpha device (pulsing 810 nm at 10 Hz). When the subject's headaches resolved after 10 days, Vielight advised him to alternate between using the Neuro Alpha and Neuro Gamma devices, while keeping the every-other-day schedule of treatments. After 3 weeks, Vielight advised the subject to continue PBM treatments every other day using just the Neuro Alpha device. This decision was based on a report that PBM treatments pulsed at 10 Hz produced the most beneficial effects in an animal model of TBI (9). Although he remained in training during the PBM treatments, the subject did not play any hockey games and did not suffer any further blows to the head.

The following neuropsychological tests were administered pre- and post-treatment: California Verbal Learning Test II (CVLT-II) (20), D-KEFS Color Word Interference test (21), Trail Making Test (TMT) (22), Digit Span subtest of the Wechsler Adult Intelligence Scale-III (WAIS-III) (23), and verbal and category (semantic) fluency (24). The following scans were acquired on a Siemens 3-Tesla Trio scanner with a 32-channel receiver head coil pre- and post-treatment: structural T1-weighted 3D Magnetization Prepared Rapid Gradient Echo image [repetition time (TR)/echo/time (TE)/inversion time (TI) = 2,500/2.98/1,100 ms, 1 × 1 × 1 mm^3^ resolution], arterial spin-labeled (ASL) magnetic resonance images (MRI) acquired with echo-planar imaging (EPI) sequence (700 ms inversion of arterial spins, 1,900 ms total transit time of spins, 100 ms tag thickness; 13 ms echo time; field of view: 256 mm, 64 × 64 matrix, 24 4-mm tick axial slices; 52 tag + control image pairs with 22.5 ms time lag between slices), and resting-state functional MRI (RS-fMRI) (8-min 12-s EPI sequence with 140 time points, 3,000 ms TR, 30 ms TE, 80° flip angle, 48 3.3-mm-thick slices, 3.3 × 3.3 × 3.3 mm^3^ resolution; 64 × 64 matrix).

FreeSurfer 6.0 and FreeSurfer's longitudinal stream (25) were used to process the structural MR images and to extract volumes from anatomical regions of interest (ROIs). Total cortical gray matter (GM) volume was derived by summing the Desikan-Killiany atlas cortical ROIs bilaterally. Total subcortical GM, thalamic, and hippocampal volumes were derived from FreeSurfer's automatic subcortical segmentation. Hippocampal subfield volumes were derived from the FreeSurfer's hippocampal subfield segmentation. The volumes from right and left hemisphere ROIs were combined.

Statistical Parametric Mapping (SPM, version 8) was used to process the ASL-MRI data, which included motion correction, aligning each ASL frame to the first frame using a rigid body transformation, and least squares fitting. Perfusion-weighted images were computed as the difference between the mean of tagged and untagged ASL data sets. To account for signal decay during acquisition and to allow for intensities in meaningful physiological units, the perfusion-weighted images were intensity scaled. After geometric distortion correction, the ASL images were aligned to structural T1-weighted images. To estimate GM perfusion and to minimize the effects of the lower perfusion in white matter on the perfusion estimates, a partial volume correction was performed using the assumption that GM perfusion is 2.5 times greater than white matter perfusion. The FreeSurfer generated anatomical ROIs were used to analyze the ASL MRI perfusion data. Mean ASL perfusion values from the cerebellum were used as a control for the meta-ROIs. Total cortical GM perfusion was derived by averaging ASL perfusion values across all the Desikan-Killiany atlas cortical ROIs bilaterally. Frontal, parietal, temporal, and occipital lobe perfusion were derived by averaging CBF values across the Desikan-Killiany ROIs that corresponded to each lobe bilaterally. Hippocampal perfusion was derived by averaging ASL perfusion values from the right and left FreeSurfer hippocampal ROIs.

CONN-fMRI Functional Connectivity toolbox version 17 (26) was used to analyze the functional connectivity data. Blood oxygen level-dependent (BOLD) signal noise from the white matter and cerebral spinal fluid was characterized with the principal component-based noise-correction “CompCor” method utilized in the CONN toolbox (27). Band-pass filtering was performed with a frequency window of 0.008–0.09 Hz and each scan was Hanning weighted (26). The ACC was chosen as a seed region to examine ROI-to-ROI functional connectivity with other brain regions. Bivariate-regression analyses were used to determine the linear association of the BOLD time series between each pair of sources in first-level analyses.

## Results

The subject's scores on tests of verbal learning and memory (CVLT-II), executive function (D-KEFS Color-Word Interference, TMT B, and verbal fluency), attention (digit span), and processing speed (TMT A, D-KEFS color naming and word reading) improved after 8 weeks of PBM treatments (see Table 2).

**Table 2:** Summary of the subject's pre- and post-treatment (Tx) neuropsychological test scores.

**California verbal learning test II**	**Pre-Tx**	**Post-Tx**	**Percentile Δ**
Trial 1 (*Z* score)	−1.5	0	7th → 50th
Trial 2 (*Z* score)	−0.5	0	32nd → 50th
Trial 3 (*Z* score)	−1	0.5	16th → 68th
Trial 4 (*Z* score)	−0.5	0	32nd → 50th
Trial 5 (*Z* score)	−0.5	0	32nd → 50th
Trials 1–5 total (standard score)	43	53	25th → 61st
Short delay free recall (*Z* score)	−0.5	−0.5	Stayed at 32nd
Short delay cued recall (*Z* score)	0	−0.5	50th → 32nd
Long delay free recall (*Z* score)	−1	−1	Stayed at 16th
Long delay cued recall (*Z* score)	0	−0.5	50th → 32nd
Total recall discriminability (*Z* score)	−1	0	16th → 50th
Immediate recall discriminability (*Z* score)	−1	0.5	16th → 68th
Delayed recall discriminability (*Z* score)	−1	0	50th → 16th
Free recall discriminability (*Z* score)	−1	−0.5	16th → 32nd
Cued recall discriminability (*Z* score)	−1	−0.5	16th → 32nd
**D-KEFS Color-Word Interference**			
Color naming (scaled score)	11	12	63rd → 75th
Word reading (scaled score)	10	11	50th → 63rd
Inhibition (scaled score)	14	16	91st → 98th
Inhibition/Switching (scaled score)	9	11	37th → 63rd
**Trail-making test**			
Part A (scaled score)	13	15	84th → 95th
Part B (scaled score)	13	14	84th → 91st
**Verbal fluency**			
FAS (raw score)	59	62	84th → 95th
Animals (raw score)	16	18	10th → 25th
**WAIS-III digit span** (scaled score)	13	17	84th → 99th

There were increases in the subject's total cortical GM (638.06–639.36 cm^3^), subcortical GM (70.73–71.10 cm^3^), and thalamic (19.11–19.19 cm^3^) volumes after PBM treatment. Although total hippocampal volume decreased (9.38–9.29 cm^3^) after 8 weeks, there were increases in volumes of the subiculum (968.37–996.92 mm^3^), CA1 (1390.78–1395.91 mm^3^), CA3 (509.17–512.72 mm^3^), hippocampal–amygdala transition zone (123.17–127.26 mm^3^), and fimbria (245.11–250.73 mm^3^) and a decrease in the volume of the hippocampal fissure (1304.47–1299.39 mm^3^).

After 8 weeks of PBM treatments, there were increases in perfusion of the subject's total cortical GM (0.53–0.64 arbitrary units), frontal lobe (0.42–0.50), temporal lobe (0.62–0.72), occipital lobe (0.71–1.15), and hippocampus (0.60–0.80).

**Figure 2:**
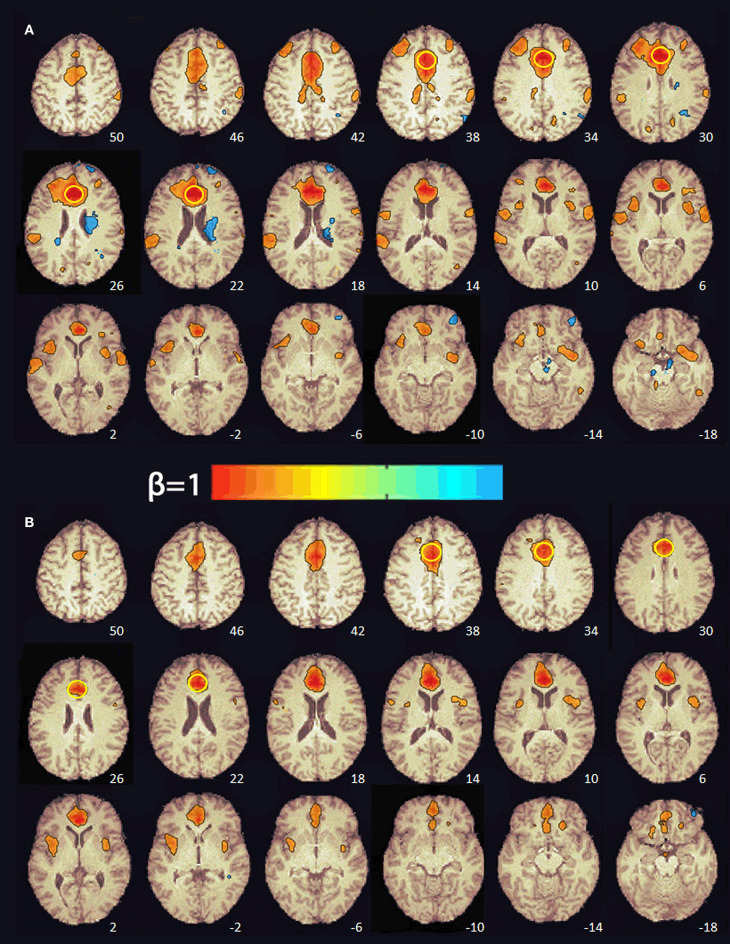
Functional connectivity maps from the pre-treatment **(A)** and post-treatment **(B)** scans showing regions functionally connected to the seed in the anterior cingulate cortex. The numbers at the bottom right indicate the *z* coordinate (mm). The yellow circle denotes the seed in the anterior cingulate cortex. The color bar indicates the beta weight of the functional connections. The maps were thresholded at β ≥ 0.4.

At baseline, multiple brain regions were functionally connected to the seed in the ACC (e.g., superior frontal gyrus, supplementary motor area, middle frontal gyrus, frontal pole, precentral gyrus, central operculum, supramarginal gyrus, posterior cingulate, parietal operculum, cuneus, lateral occipital, superior temporal gyrus, and insula; see Figure 2). After 8 weeks of PBM treatments, only the anterior insula was functionally connected to the ACC (Figure 2).

After 8 weeks of PBM treatments, the subject experienced subtle improvements in his headaches (HIT-6 score decreased from 76 to 70). We were unable to obtain additional measures post-treatment because the subject moved out of the country after treatment was completed. However, when we contacted him 14 months post-treatment, he reported that he continues to use the Vielight Neuro devices, albeit with less regularity. He has not played hockey and has not sustained any further blows to the head although he continues to train. His HIT-6 score at the 14-month follow-up was 50.

## Discussion

This report started with Vielight's attempt to help with an athlete's request to improve his “mental acuity.” The athlete's diminished mental acuity may relate to his history of concussions, for which he had previously tried nutritional supplements, acupuncture, chiropractic neurology, and hyperbaric oxygen treatment.

An estimated 1.6–3.8 million sports-related mTBIs occur in athletes annually (28–30), and cognitive dysfunction from sports-related mTBI is becoming an increasing concern (31). For example, verbal learning scores measured in the post-season were lower in college athletes who participated in contact sports compared to non-contact sport athletes (24% vs. only 3.6% with low scores) (32). Furthermore, athletes who sustained the most head impacts during the season tended to have the slowest reaction times on Immediate Post-Concussion Assessment and Cognitive Testing (ImPACT) (32). Thus, it is noteworthy that the subject's scores on tests of verbal learning and memory, executive function, attention, and processing speed improved after 8 weeks of PBM treatments.

Although different forms of the CVLT-II were administered at the two time points, we cannot discount the possibility that these improvements may, at least in part, be related to practice effect. Nor can we rule out the potential influence of a placebo effect or uneven effort. One limitation of this study is we did not formally test effort. However, certain CVLT-II measures have been used to ascertain effort (33–38). For example, CVLT long delay cued recall has been used to detect poor effort in patients with TBI (35, 39). Thus, it is notable that the subject's scores on CVLT-II short and long delay cued recall declined after 8 weeks, even though his free recall scores improved. According to the CVLT-II manual, cued recall requires the retrieval of words according to a language-based strategy. Because the subject's animal fluency scores were low at baseline (10th percentile) and improved only to the 25th percentile after treatment, it is possible that this subject has some inherent language/semantic impairment. Two other CVLT-II formulas have been used to detect effort [i.e., discriminate function (35) and logistic regression (36)], although they have been shown to misclassify individuals with lower levels of education and there were no monetary incentives for incomplete effort (40). These formulas suggest that our subject exerted incomplete effort at both time points; however, he did not have a college degree and was not involved in litigation or seeking financial compensation for alleged injuries at the time of assessments. Therefore, it is possible that his attention and concentration problems resulted in uneven effort during neuropsychological testing rather than malingering.

There have been prior reports of transcranial PBM treatments in patients with chronic TBI (11–14). Naeser et al. reported cognitive improvements in 2 chronic mTBI cases after home-based transcranial PBM treatments (11) and in 11 chronic mTBI patients who participated in an open-protocol study of transcranial PBM (12). Morries et al. reported improvements in headache, sleep disturbance, cognition, mood dysregulation, anxiety, and irritability symptoms in 10 patients with chronic TBI after transcranial treatment with a Class IV high-power NIR laser (14). Cognition appeared to improve based on return to work or improved work performance. Based on the observations of the patients, their family members, and the treating clinician, quality of life also improved in the cases (14).

Because there have been no large, controlled clinical trials of PBM for TBI, widespread acceptance of PBM as a treatment option for TBI is lacking. In this respect, neuroimaging measures may provide unbiased, objective measures of functional improvements after PBM treatment. TBI-associated cerebral atrophy is a well-documented phenomenon (41). While results in mTBI have been inconclusive, observations for moderate-to-severe TBI converge toward generalized atrophy across the entire brain, on the order of 5% per year, and focal atrophy in subcortical brain regions including the thalamus and hippocampus (41). We observed increases in the subject's total cortical GM, total subcortical GM, thalamic, and hippocampal subfield volumes. Volumetric changes in a single subject after 8 weeks should be interpreted cautiously as they are within the range of observational error. Nevertheless, it is noteworthy that animal research has shown PBM to stimulate neurogenesis and protect against cell death (4, 42–44). NIR stimulates neurite outgrowth mediated by nerve growth factor (45). In animal models of TBI, NIR (810 nm) has been shown to improve neurogenesis and synaptogenesis via increase of brain-derived neurotrophic factor (4, 8, 9, 44, 46).

Significant reductions in cerebral blood flow (CBF) and lymphatic flow, particularly in the frontal and temporal lobes, have been reported in single-photon emission computed tomography studies of chronic TBI patients relative to healthy controls (47–50). In subacute mTBI patients with no significant CT or MRI abnormalities, hypoperfusion bilaterally in the frontal lobe and in the left occipital lobe has been detected with ASL MRI, a non-invasive imaging method that uses blood water as an endogenous freely diffusible tracer to measure CBF (51). In the present case, there was increased perfusion in the frontal, temporal, and occipital lobes and the hippocampus after 8 weeks of PBM treatments. These findings are consistent with previous reports of PBM-related increases in local CBF (52), oxygen consumption (53), total hemoglobin, a proxy measure for regional CBF (54), regional CBF (13), and increased oxygenated/decreased deoxygenated hemoglobin concentrations (55).

RS-fMRI is the measure of spontaneous, correlated fluctuations in the BOLD signal that occur between functionally related brain regions when the brain is not engaged in a specific task (56). RS-fMRI has been used to identify intrinsic neural networks and to extract information about the connectivity and functionality of specific brain networks (57, 58). Previous RS-fMRI studies of mTBI patients have reported increases in both the number and the strength of connections between medial prefrontal regions (e.g., ACC) and other brain regions relative to healthy controls (59–66). For this reason, we selected the ACC as a seed region to investigate changes in functional connectivity pre- and post-PBM treatment. Before treatment, the subject's ACC was functionally connected to multiple brain regions in the frontal, parietal, temporal, and occipital cortex. After 8 weeks of PBM treatments, there was a decrease in both the number and the strength of the functional connections with the ACC. In fact, only the anterior insula, part of salience network (58), was functionally connected with the ACC. It has been hypothesized that the enhancements in functional connectivity seen in mTBI patients may reflect compensatory neural processes (63, 64). In a mouse model of TBI, Xuan et al. (44) found that neurological severity score, a measure of injury severity, and cognitive performance in the TBI mice improved over 4 weeks despite increases in the size of the TBI-induced brain lesions over the same period of time. They suggested that this occurred because the uninjured part of the mouse brain was steadily taking over more of the functions of the injured part of the brain. This evidence of neuroplasticity may be the compensatory mechanism that enhances functional connectivity in patients with mTBI. In the present case, the reduction in ACC functional connectivity after 8 weeks of PBM treatments may reflect a diminished need for compensatory processes after brain function normalized with PBM.

How does PBM promote brain recovery from TBI? Research has shown that PBM produces short-term increases in adenosine triphosphate (ATP) (67–70), blood (13, 54, 55), and lymphatic flow (71, 72); upregulates anti-apoptotic proteins (73–75), neurotrophins (43, 44, 76, 77), neurogenesis (44, 78), and synaptogenesis (44); and reduces edema (72, 79, 80), inflammation (81–84), and excitotoxicity (85). The best-studied mechanism of PBM centers on its effects in the mitochondria (86): In hypoxic or injured cells, the mitochondria's ability to produce ATP is reduced, likely because nitric oxide (NO), also produced in the mitochondria, can bind to cytochrome C oxidase (CCO), which inhibits respiration and displaces oxygen (87). When photons of light delivered during PBM are absorbed by CCO (88), NO is dissociated from CCO and mitochondrial inhibition is reversed (89). Photons of light delivered during PBM can also alter mitochondrial membrane permeability and ion flux, which can result in a brief increase in the production of reactive oxygen species (ROS) that shift the overall cell redox potential in the direction of greater oxidation, decreasing reactive nitrogen species (90) and increasing mitochondrial membrane potential. When this occurs, there is an increase in oxygen consumption, glucose metabolism, and ATP production (86). The brief increase in ROS can also change the activity of redox-sensitive transcription factors such as activator protein-1 and NF-κB (91), which, in turn, can activate signaling pathways and transcription factors that cause changes in protein expression (86).

This report has several limitations that should be acknowledged: First, the improvements observed on neuropsychological test scores after 8 weeks of PBM may have been, at least in part, influenced by practice and potentially a placebo effect. Second, effort was not formally tested. However, examination of certain CVLT-II measures suggests that the subject's effort on neuropsychological testing may have been uneven, possibly because of his difficulty maintaining attention and concentration. In the search for the most effective PBM regime, there were non-systematic changes in the PBM device and the combination of devices that the subject used. Because the subject's motivation for trying PBM was to improve mental acuity, Vielight initially recommended the Neuro Gamma device (pulsing NIR at 40 Hz) based on another report of the Neuro Gamma's ability to improve cognition (19). However, the subject developed mild headaches after 1 week, which resolved after he switched to the Neuro Alpha device (pulsing NIR at 10 Hz). In this regard, it is noteworthy that PBM at 10 Hz has precedent of helping to alleviate TBI symptoms (9) and pulsing PBM has been shown to modulate brain oscillations in frequency-specific ways that could influence brain functions (66). Other limitations include monitoring the subject's adherence to the PBM treatment solely through self-reports, not controlling for physical activity or diet during the treatment period, and not having measures of the variance of the imaging outcomes in healthy controls. The changes that we observed after 8 weeks in this single case may be subject to observational error. Finally, this report would have been strengthened had we tested other biomarkers that might support our hypothesis of neuroplasticity as etiology of neuroimaging changes. These limitations notwithstanding, the present case report, along with other published studies (11), suggest that it is possible for individuals with histories of concussion or TBI to self-administer PBM therapy at home. Together with previous reports of the beneficial effects of PBM in chronic TBI patients (11–14) and in preclinical studies of acute TBI (4–9), this single, sports-related concussion case suggests that larger, controlled trials of PBM for TBI and additional research on the optimal PBM treatment parameters for TBI are warranted.

## Data Availability Statement

The datasets generated for this study are available on request to the corresponding author.

## Ethics Statement

Ethical review and approval was not required for the study on human participants in accordance with the local legislation and institutional requirements. The patients/participants provided their written informed consent to participate in this study. Written informed consent was obtained from the individual(s) for the publication of any potentially identifiable images or data included in this article.

## Author Contributions

LC: conceptualization of neuropsychological and neuroimaging measures, acquisition of neuropsychological data, formal data analysis and interpretation, and writing original draft. CB: acquisition and pre-processing of neuroimaging data. LL: determined parameters of the PBM devices. CB, MK, and LL: contributed to the content of and final approval of the manuscript. All authors contributed to the article and approved the submitted version.

## Conflict of Interest

MK and LL are employees of Vielight, Inc., the manufacturer of the devices used in this report. The remaining authors declare that the research was conducted in the absence of any commercial or financial relationships that could be construed as a potential conflict of interest.
