# Supplementary material for: Changes in Brain Function and Structure After Self-Administered Home Photobiomodulation Treatment in a Concussion Case
Source: Front Neurol. 2020 Sep 8;11:952. doi: 10.3389/fneur.2020.00952 (PMC7509409; doi:10.3389/fneur.2020.00952)
Supplement: Supplementary file 1 [file Table_1.DOCX]

**Supplementary Table. Volumes and perfusion values from Freesurfer ROIs**

| **Brain Region** | **TP1 volume^a^** | **TP2 volume^a^** | **TP1 perfusion^b^** | **TP2 perfusion^b^** |
| --- | --- | --- | --- | --- |
| **Frontal Lobe** |  |  |  |  |
| Superior frontal | 56.46 | 56.37 | 0.35 | 0.27 |
| Frontal pole | 2.66 | 2.69 | 0.28 | 0.65 |
| Medial orbitofrontal | 15.24 | 15.30 | 0.41 | 0.52 |
| Rostral anterior cingulate | 6.96 | 7.00 | 0.34 | 0.48 |
| Caudal anterior cingulate | 5.61 | 5.53 | 0.38 | 0.49 |
| Precentral | 34.15 | 33.98 | 0.37 | 0.36 |
| Rostral middle frontal | 42.42 | 42.91 | 0.35 | 0.45 |
| Caudal middle frontal | 14.44 | 14.62 | 0.50 | 0.40 |
| Parsoperculum | 15.12 | 15.12 | 0.44 | 0.49 |
| Parstriangularis | 10.84 | 10.89 | 0.56 | 0.38 |
| Parsorbitalis | 6.79 | 6.88 | 0.61 | 0.75 |
| Lateral orbitofrontal | 21.15 | 21.11 | 0.39 | 0.69 |
| **Parietal Lobe** |  |  |  |  |
| Paracentral | 10.28 | 10.34 | 0.28 | 0.44 |
| Posterior cingulate | 7.83 | 7.91 | 0.49 | 0.51 |
| Isthmus of the cingulate | 6.81 | 6.90 | 0.83 | 0.72 |
| Precuneus | 24.96 | 25.05 | 0.53 | 0.72 |
| Postcentral | 24.74 | 34.75 | 0.48 | 0.41 |
| Superior parietal | 34.42 | 41.57 | 0.56 | 0.34 |
| Inferior parietal | 41.35 | 41.28 | 0.62 | 0.47 |
| Supramarginal | 28.35 | 28.28 | 0.52 | 0.49 |
| **Temporal Lobe** |  |  |  |  |
| Transverse temporal | 2.55 | 2.55 | 0.95 | 0.84 |
| Bank of the superior temporal sulcus | 6.20 | 6.17 | 0.59 | 0.55 |
| Superior temporal | 29.25 | 29.26 | 0.58 | 0.67 |
| Middle temporal | 34.43 | 34.46 | 0.63 | 0.62 |
| Inferior temporal | 27.89 | 27.83 | 0.59 | 0.57 |
| Enthorinal cortex | 4.85 | 4.72 | 0.66 | 0.68 |
| Parahippocampal gyrus | 4.84 | 4.86 | 0.68 | 1.02 |
| Fusiform gyrus | 24.82 | 24.99 | 0.58 | 1.15 |
| Temporal pole | 6.47 | 6.40 | 0.30 | 0.34 |
| **Occipital Lobe** |  |  |  |  |
| Lateral occipital cortex | 34.68 | 35.01 | 0.74 | 0.82 |
| Cuneus | 9.88 | 10.14 | 0.65 | 1.24 |
| pericalcarine | 7.24 | 7.16 | 0.65 | 1.12 |
| Lingual gyrus | 17.57 | 17.65 | 0.79 | 1.43 |
| **Other** |  |  |  |  |
| Insula | 16.66 | 16.40 | 0.49 | 0.60 |
| Total subcortical GM | 846.19 | 848.54 | 0.33 | 0.43 |
| Thalamus | 19.11 | 19.19 | 0.44 | 0.25 |
| Hippocampus | 9.38 | 9.29 | 0.60 | 0.80 |
| **Hippocampal Subfields^c^** |  |  |  |  |
| Parasubiculum | 133.17 | 128.51 |  |  |
| Presubiculum | 712.59 | 699.11 |  |  |
| Subiculum | 968.37 | 996.92 |  |  |
| CA1 | 1390.78 | 1395.91 |  |  |
| CA3 | 509.17 | 512.72 |  |  |
| CA4 | 703.84 | 689.05 |  |  |
| GC-DG | 747.83 | 735.41 |  |  |
| HATA | 123.17 | 127.27 |  |  |
| fimbria | 245.11 | 250.73 |  |  |
| Molecular layer | 1304.47 | 1299.39 |  |  |
| Hippocampal fissure | 358.04 | 349.93 |  |  |
| Hippocampal tail | 1350.34 | 1332.17 |  |  |

^a^in cm^3^, ^b^in arbitrary units, ^c^in mm^3^
